# Endocrinology and physiology of pseudocyesis

**DOI:** 10.1186/1477-7827-11-39

**Published:** 2013-05-14

**Authors:** Juan J Tarín, Carlos Hermenegildo, Miguel A García-Pérez, Antonio Cano

**Affiliations:** 1 Department of Functional Biology and Physical Anthropology, Faculty of Biological Sciences, University of Valencia, Burjassot, Valencia 46100, Spain; 2 Research Unit-INCLIVA, Hospital Clínico de Valencia, Faculty of Medicine, University of Valencia, Valencia 46100, Spain; 3 Department of Physiology, Faculty of Medicine, University of Valencia, Valencia 46100, Spain; 4 Department of Genetics, Faculty of Biological Sciences, University of Valencia, Burjassot, Valencia 46100, Spain; 5 Department of Pediatrics, Obstetrics and Gynecology, Faculty of Medicine, University of Valencia, Valencia 46100, Spain; 6 Service of Obstetrics and Gynecology, University Hospital Dr. Peset, Valencia 46017, Spain

**Keywords:** Catecholamines, Hypothalamus-pituitary-ovarian axis, Major depression disorder, Polycystic ovarian syndrome, Sympathetic nervous system

## Abstract

This literature review on pseudocyesis or false pregnancy aims to find epidemiological, psychiatric/psychologic, gynecological and endocrine traits associated with this condition in order to propose neuroendocrine/endocrine mechanisms leading to the emergence of pseudocyetic traits. Ten women from 5 selected studies were analyzed after applying stringent criteria to discriminate between cases of true pseudocyesis (pseudocyesis vera) versus delusional, simulated or erroneous pseudocyesis. The analysis of the reviewed studies evidenced that pseudocyesis shares many endocrine traits with both polycystic ovarian syndrome and major depressive disorder, although the endocrine traits are more akin to polycystic ovarian syndrome than to major depressive disorder. Data support the notion that pseudocyetic women may have increased sympathetic nervous system activity, dysfunction of central nervous system catecholaminergic pathways and decreased steroid feedback inhibition of gonadotropin-releasing hormone. Although other neuroendocrine/endocrine pathways may be involved, the neuroendocrine/endocrine mechanisms proposed in this review may lead to the development of pseudocyetic traits including hypomenorrhea or amenorrhea, galactorrhea, diurnal and/or nocturnal hyperprolactinemia, abdominal distension and apparent fetal movements and labor pains at the expected date of delivery.

## Background

According to the fifth edition of Diagnostic and Statistical Manual of Mental Disorders (DSM-5) [1], pseudocyesis [from the Greek words *pseudes* (false) and *kyesis* (pregnancy); a.k.a. false, imaginary, simulated, phantom, hysterical or spurious pregnancy] is a rare disorder with characteristic somatic features. It is included in the *Not Elsewhere Classified* section of *Somatic Symptom Disorders*, a group of disorders that typically present first in non-psychiatric settings characterized predominantly by somatic symptoms or concerns that are associated with significant distress and/or dysfunction. The fact that it is included in a *Not Elsewhere Classified* section means that it is in a category by itself, different from other *Somatic Symptom Disorders* such as *Functional Neurological Disorder* (previously, *Conversion Disorder*).

Pseudocyesis is defined by the DSM-5 as *a false belief of being pregnant that is associated with objective signs and reported symptoms of pregnancy, which may include abdominal enlargement, reduced menstrual flow, amenorrhea, subjective sensation of fetal movement, nausea, breast engorgement and secretions, and labor pains at the expected date of delivery*.

Cases of pseudocyesis nowadays are found more frequently in rural undeveloped countries where women usually are not examined by a physician or a midwife until they are in labor or seek medical aid [2,3]. In developed countries, women visit obstetricians in the first trimester of pregnancy who have more accurate means of diagnosis including pregnancy tests and ultrasonographic examinations. These diagnostic procedures may help pseudocyetic women to be convinced of their non-pregnant condition, that usually leads to resolution of pseudocyesis within minutes or even seconds. Furthermore, women from developed countries in general are more educated and sophisticated, and their emotional conflicts result in a more profound, refined mode of expression than that of pseudocyesis (cited by Pawlowski and Pawlowski [2]).

Table 1 shows the incidence of pseudocyesis registered in several USA and African hospitals. The relative high incidence of pseudocyesis found in these studies, except for Boston Hospitals [4], is likely due to the importance that most African black cultures give to fertility together with low educational background [3,6] and sterility problems [5,7]. For instance, in the Igbo culture of southeast Nigeria, married women wish to become pregnant because pregnancy and childbirth confirm womanhood and secure the woman’s place in her husband family. This societal pressure may precipitate pseudocyesis as a psychological defense to intense stress if the woman proves to be infertile [3]. Recently, it has been reported that African-American women perceive higher average social messages to have children than White women (social messages were assessed in a national random-digit dial telephone survey via two items: *it is important to my partner that we have children*; and *it is important to my parents that I have children*) [8].

**Table 1:** Incidence of pseudocyesis

**Frequency**	**Location**	**Race/Ethnicity**	**Reference**
1/22000 births	Boston Hospitals, USA	?	[4]
1/344 of newly booked expectant mothers	St. Vincent’s Hospital Ndubia, Ebonyi State, Nigeria	African black	[3]
1/250 maternity clinic admissions (24 out of 27 patients had tried to conceive without success for 2 to 17 years)	Jefferson Hospital, Philadelphia, USA	85% (23/27) African-American black; 15% (4/27) white	[5]
1/200 births	Baragwanath Hospital, South Africa	African black	[6]
1/160 women who had previously being investigated and managed for reproductive failure	Wad Medani Teaching Hospital, Sudan	African black	[7]

We should emphasize that the definition of pseudocyesis contrasts with delusion of pregnancy, deceptive or simulated pregnancy and erroneous pseudocyesis (for review, see O’Grady and Rosenthal [9]). Delusion of pregnancy is a *Somatic Type* (i.e., delusion that the individual has some general medical condition) *Delusional Disorder* classified by the DSM-5 within *Schizophrenia Spectrum and Other Psychotic Disorders*. It refers to the false belief of being pregnant in the absence of physical sings suggestive of pregnancy that may be experienced by psychotic women [10-12] and men [13,14]. According to the DSM-5, the disorder is not a consequence of the direct physiological effects of a substance (e.g., a drug of abuse, a medication) or a general medical condition. Although at first glance it appears that it is easy to distinguish between true pseudocyesis (pseudocyesis vera) and delusion of pregnancy, sometimes delusions of pregnancy present along with physical traits of pseudocyesis [15-18] making difficult to perform a differential diagnosis.

A deceptive or simulated pregnancy is a *Factitious Disorder Imposed on Self* classified by the DSM-5 within the category of *Somatic Symptom Disorders*. In this case, the woman acknowledges being pregnant knowing that it is not true ([19,20]; for review, see O’Grady and Rosenthal [9]).

Finally, erroneous pseudocyesis refers to cases when the woman erroneously misinterprets symptoms suggestive of pregnancy, including amenorrhea, galactorrhea and/or abdominal enlargement, resulting from either organic diseases (e.g., hormone-secreting tumor [21-23], alcoholic liver disease [24], cholecystitis [25], urinary tract infection complicated by urine retention [26]) or exposure to a substance (e.g., a medication [27-30]).

Whereas most studies have presented pseudocyesis cases as medical curiosities, a few studies have focused their attention on ascertaining the neuroendocrine/endocrine mechanism underlying this condition. These studies, however, have not reported consistent changes in plasma levels of prolactin (PRL), follicle stimulating hormone (FSH), luteinizing hormone (LH), growth hormone (GH), estradiol (E_2_), progesterone (P) and testosterone (T) (for reviews, see Small [31] and O’Grady and Rosenthal [9]). The lack of consistency among studies is not at all surprising if we take into account the small sample sizes and the different endocrine methodologies (e.g., different blood sampling frequency or type of assay for measuring hormones) used in these studies, as well as the ambiguous or null psychiatric/psychologic and/or diagnostic criteria applied to define and discriminate between cases of pseudocyesis vera versus delusional, simulated or erroneous pseudocyesis.

In this review, we aim to find epidemiological, psychiatric/psychologic, gynecological and endocrine traits associated with pseudocyesis vera in order to propose neuroendocrine/endocrine mechanisms leading to the development of pseudocyetic traits.

## Methods

A review of the current and older literature on studies on human pseudocyesis was performed. The review was based on publications up to January 2013 identified by PubMed database searches using the following key words: pseudocyesis and women. In addition, a hand search was done to explore the references cited in the primary articles. Only studies in which women met the DSM-5 definition of pseudocyesis (see above) and provided clear evidence that women did not suffer from simulated or erroneous pseudocyesis were selected for entering into the study. Furthermore, in order to exclude cases of delusion of pregnancy and focus exclusively on pseudocyesis vera, only studies in which a psychiatric/psychologic evaluation provided proof that women were non-psychotic were included in the study (Table 2). Two exceptions, however, were allowed: (1) a woman that refused psychiatric evaluation but her hormonal responses to thyroid-stimulating hormone (TRH) stimulation and dexamethasone suppression tests were similar to those seen in major depressive disorders [32]. This woman was entered into the study because most (8 out of 10 women) of the pseudocyetic women included in Table 2 suffered from mild to major depression; and (2) an unidentified member of a cohort of 5 women that was not psychologically evaluated for unreported reasons [33]. This woman was included in Table 2 because the potential bias that her inclusion may introduce in the analysis, if the woman suffered from delusion of pregnancy, is relatively small. In Table 3 we provide data from the studies excluded from the study that did not meet the inclusion criteria.

**Table 2:** Epidemiological, psychiatric/psychologic, gynecological and endocrinological traits of 10 pseudocyetic women analyzed in 5 studies that fulfilled the inclusion criteria laid down in the Methods section

**N**° **of women**	**Women’s age (y)**	**Psychiatric/psychologic traits**	**Gynecological traits**	**LH^a^**	**FSH**	**LH/FSH^a^ ratio**	**T^b^**	**E_2_**	**P**	**GH**	**PRL^c^**	**Cortisol or DHEAS**	**Response to inhibitors of the pituitary-adrenal axis**	**Response to GnRH**	**TSH and/or response to TRH^e^**	**Response to dopamine agonists (bromocriptine, apomorphine and L-DOPA)^f^ or opiate antagonists (naloxone)^g^**	**Reference**
1	16	Mild depression	Amenorrhea (9 months) and galactorrhea	Elevated with increased frequency of pulses (mid-cycle surge levels)	Within normal range	≈ 5.7	--	Within the mid-follicular phase range	slightly higher than the follicular phase range	Normal diurnal episodic secretion	Distinctly elevated (similar to the mid-cycle surge)	--	--	--	Basal TSH within normal range	--	[34]
1	39	Depression	Amenorrhea (5 months) and galactorrhea	≈ 0.2 IU/L	≈ 14 IU/L	≈ 0.1	--	Within the follicular phase range	Within the follicular phase range	--	Within the normal range	--	--	Normal increase in LH and FSH after GnRH	Normal increase in TSH and PRL, and paradoxical increase in GH (normal basal levels of TSH)	Normal decrease in PRL after bromocriptine and normal decrease in PRL and blunted increase in GH after apomorphine	[35]
1	27	Likely unipolar depressive disorder	Amenorrhea (9 months) and galactorrhea	Within follicular phase range	Within follicular phase range	2.0	Within mid-follicular phase range	Within the mid-follicular phase range	Within mid-follicular phase range	--	Within the normal range	Minimally elevated DHEAS levels	Normal suppression of A.M. cortisol but ACTH remained elevated after overnight low-dose (1 mg) dexamethasone^d^	--	Small increase in TSH, normal increase in PRL and paradoxical increase in GH (normal basal levels of TSH and T_4_)	--	[32]
5	19, 29, 33, 34, 35	4 women with depression and hysteria	Amenorrhea (4–15 months) and galactorrhea	Within normal range	Within normal range	8.0, 6.6, 2.7, 2.9, 4.4	--	Within normal range (4 women)	1 woman within the follicular phase range, and 4 women slightly higher than the follicular phase range	--	Slightly elevated (4 women) and within the normal range (1 woman)	--	--	--	--	No increase in LH or PRL after naloxone	[33]
1	30	Histrionic personality suffering from major depressive disorder and borderline personality disorder	Amenorrhea (7 months) and galactorrhea	Elevated with frequency of pulses higher than late-follicular phase	Within normal range	≈ 11.3	Consistently elevated	Elevated (late-follicular phase levels)	Not elevated (late-follicular phase levels)	Decreased nocturnal peaks of GH	Normal levels with greater release during sleep than during daytime	Normal cortisol levels rising during sleep and reaching a maximum at 08.00-09.00 h	--	--	--	Decrease in PRL levels and no increase in GH after L-DOPA	[16]
1	38	Histrionic and hypochondriac personality	Amenorrhea (> 5 months) and galactorrhea	Normal with frequency of pulses similar to late-follicular phase	Within normal range	≈ 2.9	Consistently elevated	Elevated (late-follicular phase levels)	Not elevated (late-follicular phase levels)	Decreased nocturnal peaks of GH	Normal levels with greater release during sleep than during daytime	Normal cortisol levels rising during sleep and reaching a maximum at 08.00-09.00 h	--	--	--	Decrease in PRL and no increase in GH after L-DOPA	[16]

^a^Despite discrepancies among studies in levels of LH, all the studies included in Table 2 excluding one [35] evidenced LH/FSH ratios higher than 2.0. Moreover, the two studies [16,34] that measured and analyzed pulsatile patterns of LH in 3 women reported increased (“higher than or similar to the late follicular phase” [16] or “similar to the mid-cycle surge” [34]) frequency of pulses. It is important to stress that these women exhibited high levels of E_2_ similar to those found at the late-follicular phase [16], and high levels of PRL similar to those observed mid-cycle just before ovulation [34]. As the frequency of LH pulses increases during the pre-ovulatory period in women with normal menstrual cycles (for review, see McCartney et al. [36]), these data suggest that the 3 pseudocyetic women with high frequency of pulses were in the pre-ovulatory phase of the menstrual cycle at the time blood samples were collected. However, it is very unlikely that the 3 pseudocyetic women restarted ovarian cyclicity just before going to the doctor after being amenorrheic for a long (5 to 7 months). On the contrary, women may have restarted ovarian cyclicity after being informed of her non-pregnant state. In fact, 2 women menstruated 3 [16] and 2 [34] weeks, respectively, after being told that they were not really pregnant (basal hormone determinations were performed before women were informed of their non-pregnant state). Of note, in the woman that menstruated 2 weeks after disclosure of diagnosis, the distended abdomen disappeared within 30 min and the basal levels of LH, PRL and E_2_ decreased shortly after being informed of her false pregnancy. All these circumstances support the notion that the higher frequency of LH pulses evidenced in these women was indeed due to their pseudocyetic condition rather than to spontaneous resumption of ovarian cyclicity before going to the doctor.

^b^In these studies, total serum T concentrations were measured by direct radioimmunoassay, a method that suffers from a number of serious problems. This assay often overestimates T concentrations and has limited accuracy at T < 10.4 nmol/L at concentrations typically found in women [37]. Moreover, total serum T levels vary across the menstrual cycle. In normal cycling women, T levels are relatively high at the mid-follicular phase, peaking on the day of LH peak [38,39]. As mentioned above, despite the 2 women analyzed by Starkman et al. [16] displayed LH pulse characteristics and E_2_ and P levels similar to those found at the late-follicular phase, these traits were likely associated to their pseudocyetic condition. Therefore, the “consistently elevated” T levels reported by Starkman et al. [16] cannot be ascribed to women being at the late-follicular phase (when serum T levels are highest). In fact, the 2 women exhibited T concentrations (5.2 and 4.2 nmol/L, respectively) much higher than those observed in normal cycling women at mid-cycle using direct radioimmunoassay (≈3.3 nmol/L [38]), isotope dilution-liquid chromatography-tandem mass spectrometry (1.7 nmol/L [39]) or automated delayed one step chemiluminescent microparticle immunoassay (2.0 nmol/L [39]).

^c^All the women included in Table 2 had galactorrhea despite 50% of them being normoprolactinemic. The absence of correlation between the presence of galactorrhea and levels of PRL may be explained by the fact that blood sample collections were performed in the morning in the majority of the studies. Such a sampling schedule may have concealed the possible occurrence of transient nocturnal hyperprolactinemia associated with galactorrhea and diurnal normoprolactinemia as reported in infertile normoprolactinemic women [40]. In fact, the only study in Table 2 that determined PRL levels in 2 pseudocyetic women during the night [16] reported rising levels during sleep but normal levels during the day. Although the pattern of pituitary PRL release in women with normal cycles follows this circadian rhythm (for review, see Bouilly et al. [41]), the nocturnal PRL concentrations (≈869.6 to ≈ 2174.0 pmol) reported by Starkman et al. [16] are more consistent with those found at night in women with nocturnal hyperprolactinemia, galactorrhea and diurnal normoprolactinemia (608.7 to 1130.4 pmol) than with those displayed by women with normal cycles (347.8 to 608.7 pmol) [40]. Interestingly, the galactorrhea exhibited by women with nocturnal hyperprolactinemia and diurnal normoprolactinemia improved in ≈ 90% (8/9) of cases after treatment with the dopamine agonist bromocriptine [40]. These facts suggest that normoprolactinemic pseudocyetic women (50% in the present review) may have occult hyperprolactinemia due to a deficit in brain dopamine activity.

^d^The discrepancy between the cortisol and adrenocorticotropin hormone (ACTH) response to dexamethasone may be due to the low sensitivity and specificity of the ACTH assay used in this study [42]. However, the fact that after resolution of pseudocyesis both ACTH and cortisol levels decreased after dexamethasone administration suggests that the discordant response observed by Ayers and Seiler [32] was associated with pseudocyesis.

^e^After resolution of pseudocyesis, women had no GH response to TRH [32,35] and the blunted or reduced thyroid-stimulating hormone (TSH) response reverted to normal [32] which occurs in patients with major depression after clinical recovery [43,44]. The mechanism of the paradoxical response of GH to TRH has not been yet elucidated (cited by Arita et al. [45]). However, some authors have speculated about the occurrence of a disruption of the normal neuroendocrine regulatory mechanisms and/or alteration of the cellular receptors of the somatotroph cells in adenomatous tissue [46].

^f^Dopamine stimulates hypothalamic GH-releasing hormone (GHRH) release [47] but inhibits the high intrinsic PRL secretory activity of the pituitary lactotrophs as well as PRL gene expression and lactotroph proliferation [48]. However, the elevated levels of T displayed by pseudocyetic women may decrease the response of GH to apomorphine and L-3,4-dihydroxyphenylalanine (L-DOPA) since T directly stimulates somatostatin [a.k.a. GH-inhibiting hormone (GHIH) or somatotropin release-inhibiting factor (SRIF)] release from the periventricular nucleus of the hypothalamus (for review, see Spiliotis [49]). It should be emphasized that the decreased GH secretion after apomorphine evidenced by Tulandi et al. [35] reverted to normal after resolution of pseudocyesis.

^g^It is known that endogenous opioid peptides inhibit simultaneously LH and PRL secretion mediated by steroid-dependent suppression of hypothalamic release of GnRH (for review, see Yen et al. [50]). In fact, women with normal cycles exhibit a positive LH and PRL response to naloxone during the late-follicular and mid-luteal phases (when E_2_ and P levels are relatively high) but not in the early-follicular phase of the menstrual (for review, see Yen et al. [50]). Note that although the pseudocyetic women analyzed by Devane et al. [33] had P levels “slightly higher” [mean ± standard error of the mean (SEM): 8.9 ± 2.5 nmol/L] than the normal follicular phase range (<3.2 nmol/L [33]), levels of E_2_ (200.8 ± 47.7 pmol/L) were within the early follicular range (73.4 to 212.2 pmol/L [51]). Thus, the presence of relatively low levels of E_2_ may explain the absence of response of LH and PRL to naloxone evidenced by Devane et al. [33] such as occurs in women with normal cycles in the early-follicular phase cycle or in hypogonadal women (for review, see Yen et al. [50]) and, therefore, it does not support a role for brain opioid peptides in pseudocyesis.

**Table 3:** Epidemiological, psychyatric/psychologic, gynecological and endocrinological traits of pseudocyetic women from studies discarded because they did not meet the inclusion criteria laid down in the Methods section

**N**° **of women**	**Women’s age (y)**	**Psychiatric/ psychologic traits**	**Gynecological traits**	**LH**	**FSH**	**LH/FSH ratio**	**T**	**E_2_**	**P**	**GH**	**PRL**	**Cortisol or DHEAS**	**Response to GnRH or EB^a^**	**TSH and/or response to TRH**	**Response to dopamine agonists^b^ or antagonists^c^**	**Reference**
2	19, and 33	--	Amenorrhea (6 and 8 months, respectively), galactorrhea, and presence of a proliferative endometrium	Total gonadotropin activity below the normal limits found in normal cycling women	Total gonadotropin activity below normal limits found in normal cycling women	--	--	Total estrogen activity below normal range found in normal cycling women	--	--	Distinctly elevated	--	Marked increase in LH and, to a lesser extent, FSH after GnRH	Marked increase in PRL and normal increase in TSH (basal levels of total T_4_, free T_4_ and thyroid binding globulins within normal range)	--	[52]
6	38.0 ± 2.5^c^	--	Amenorrhea and galactorrhea	Slightly elevated but within normal range	Within normal range	11.5	--	--	--	--	Markedly elevated	--	--	--	--	[53]
6	42.8 ± 6.3^c^	--	Amenorrhea without galactorrhea	Markedly elevated	Elevated	21.3	--	--	--	--	Within normal range	--	--	--	--	[53]
2	40 and 26	No treatment with tranquilizers in the past	Amenorrhea (3 and 7 months, respectively), galactorrhea, and recurrence of pseudocyesis^e^	Normal	Normal	0.6 and 2.1	--	Within the follicular phase range	Within the follicular phase range	--	Within the normal range	--	Marked or normal increase in LH and normal increase in FSH after GnRH	Marked increase in PRL, normal increase in TSH and paradoxical increase in GH after TRH (normal basal levels of TSH and T_4_, and normal fasting blood glucose levels)	Blunted increase of GH (PRL was not measured) after apomorphine^a^ in one woman and no response of GH and normal decrease in PRL after bromocriptine^a^ in the other woman	[54]
1	16	--	Amenorrhea (5 months) without galactorrhea, and presence of a proliferative endometrium	Elevated (within the climacteric range)	Within the follicular phase range	2.9	--	Within the follicular phase range	Within the follicular phase range	--	Within the normal range	--	Normal increase in LH and FSH after GnRH; and normal positive feedback of LH after EB	Normal PRL and TSH increase after TRH (normal basal levels of TSH)	Normal increase in PRL after metoclopramide^b^	[55]
1	17	--	Amenorrhea (8 months) without galactorrhea, and the presence of a proliferative endometrium	Within the follicular phase range	Within the follicular phase range	0.9	--	Within the follicular phase range	Within the follicular phase range	--	Within the normal range	--	Normal increment in LH and FSH after GnRH, and normal positive feedback of LH after EB	Normal PRL and TSH increase after TRH (normal basal levels of TSH)	Normal increase of PRL after metoclopramide^b^	[55]
5	19-35 (median 22)	Women with a psychiatric condition that precluded informed consent were excluded	Amenorrhea (5.5-12.5 months), galactorrhea (3 women), and hirsutism (3 women, one of them with clitoromegaly)	Below the upper normal limit (<20 IU/L) with either low-normal or definitely low pulse amplitudes (early-follicular phase)	Within the follicular phase range	1.2	Elevated in 3 women. Another women with mildly elevated FAI^f^	Within the early-follicular range	Within the early-follicular range.	--	Normal levels with greater release during sleep than during daytime (1 women displayed high PRL levels)	--	--	--	--	[56]
9	24.9 ± 2.2^d^	--	Amenorrhea (3–12 months) and galactorrhea (8 women)	Within the follicular phase range	Within the follicular phase range	1.1	--	6 women with lower levels than the follicular phase range	2 women with luteal phase levels	5 women exhibited impaired GH responses to hypoglycemia (4 of them also displayed E_2_ deficiency)	Within follicular phase range	Normal responses of cortisol to hypoglycemia (peak responses exceeded 600 nmol/L)	4 women with exaggerated LH response (2 of them also with exaggerated FSH response) after GnRH	Normal PRL and TSH increase after TRH (1, 1 and 2 women had exaggerated PRL, GH and TSH responses, respectively)	Decrease in PRL. In 4 women, no increase in GH after L-DOPA^a^ (3 of these women displayed also impaired GH response to hypoglycemia)	[57]

^a^Estradiol benzoate.

^b^Dopamine agonist.

^c^Dopamine antagonist.

^d^Mean ± SEM.

^e^This was the 40-year-old woman with 3 months of amenorrhea and a LH: FSH ratio of 0.6 [the same woman analyzed by Tulandi et al. [35] (see Table 2)].

^f^Free androgen index.

The chronological age of pseudocyetic women included in Table 2 ranged from 16 to 39 years. Eight women suffered from mild to major depression, one woman was not psychologically evaluated for unreported reasons [33] and another did not meet the criteria for depression disorder but exhibited a histrionic and hypochondriac personality [16]. As the majority of the pseudocyetic women suffered from mild to major depression and 3 [32-35] out of the 5 studies suggested that the endocrinological traits of pseudocyetic women resemble those of polycystic ovarian syndrome (PCOS), data from pseudocyetic women were qualitatively compared with those reported for PCOS and major depressive disorder.

PCOS is a heterogeneous disease characterized by hyperandrogenemia (predominantly of ovarian origin: the ovaries produce up to 60% of androgens, while the remaining 40% is of adrenal origin), hirsutism, oligo- or amenorrhea and anovulation, and is frequently associated with hyperinsulinemia, insulin resistance syndrome, increased cardiovascular risk and diabetes mellitus (for reviews, see McCartney et al. [36], Rotterdam ESHRE/ASRM-Sponsored PCOS Consensus Workshop Group [58] and Burt Solorzano et al. [59]). Note as well that there are many subgroups of depression differing from each other in their neuroendocrine/endocrine traits. Thus, for the sake of homogeneity, we chose the well-characterized major depressive disorder group as a reference group.

It is important to mention that the studies included in Table 2 used different endocrine methodologies (e.g., different blood sampling frequency or type of assay for measuring hormones). For this reason, the absolute hormonal concentrations reported in these studies cannot be compared among studies. Instead, Table 2 and Table 3 provide the authors’ original terminology that was used to describe whether hormonal levels were “elevated”, “low” or “within normal range” when compared to control data from either the own authors’ laboratory [33,34] or from other contemporary sources [16,32,35].

### Similarities and differences among pseudocyesis, PCOS and major depressive disorder

Table 4 shows a summary of the traits that characterize pseudocyesis, PCOS and major depressive disorder according to the variables included in Table 2. The comparative analysis shows that pseudocyetic women share many endocrine traits with PCOS and major depressive disorder, although these traits are more akin to PCOS than to major depressive disorder. Note that the fact that pseudocyetic women share many endocrine traits with PCOS does not necessarily mean that pseudocyetic women suffer from PCOS (we are not aware of any study reporting the presence of polycystic ovaries in pseudocyetic women). As mentioned above, pseudocyesis is a psychiatric condition. It is not a consequence of either organic diseases (including PCOS) or exposure to a substance.

**Table 4:** **Comparative analysis of pseudocyesis, PCOS and major depressive disorder traits according to the variables included in Table **2^**a**^

**Variables included in Table **2	**Pseudocyesis**	**PCOS**	**Major depressive disorder**
Amenorrhea	**Yes**	**Yes **[36,59]	No [60]
Galactorrhea	**Yes**	**Yes^b^**	No [60]
LH/FSH ratio	**Increased**	**Increased**^**c**^	?
Frequency of LH pulses	**Increased**	**Increased**^**c**^	Decreased frequency and dysrhythmic pulses [60]
T levels	**Elevated**	**Elevated**[61]	Elevated [62]
E_2_ levels	**Within the (broad) normal ranges**	**Within the (broad) normal ranges**[63]	Decreased levels in the follicular phase^d^
P levels	**Within or slightly higher than the follicular phase range**	**Within either the follicular phase range or the lutheal phase range**^**e**^	Normal P levels on the first day after menstruation [62] and higher P levels in the luteal phase [64]
GH levels	**Decreased nocturnal peaks and normal diurnal episodic secretion**	Low, normal or elevated levels with impaired diurnal secretion [65]	**Decreased nocturnal secretion **[66]
PRL levels	**Normal (putative occult hyperprolactinemia), slightly elevated or distinctly elevated**	**Hyperprolactilemia (10-15% of PCOS women) **[36]	Normal [62]
DHEAS levels	**Minimally elevated**	**Elevated (20-33% of PCOS women **[67]**)**	Normal [68]
Cortisol levels	**Normal**	**Normal **[67]	Disturbed diurnal rhythms, higher evening levels and accentuated post-awakening surge [69]
Response of ACTH to dexamethasone	**Escape from suppression**	?	**Some individuals escape from suppression **[42]
Response of cortisol to dexamethasone	**Suppression**	**Suppression **[70]	Some individuals escape from suppression [42]^f^
Response of LH and FSH to GnRH	**Normal increase**	Higher increase in LH and normal increase in FSH [71]	**Normal increase**[62]
Response of GH to TRH	**Paradoxical increase**	**Paradoxical increase (≈42% of PCOS women **[46]**)**	**Paradoxical increase **[43]
Response of TSH to TRH	**Normal or small increase**	?	**Small increase **[43,44]
Response of GH to dopamine agonists	**Deficient or reduced increase**	**Deficient or reduced increase **[72,73]^**g**^	**Normal **[74]**or reduced increase**[75-77]
Response of LH and/or PRL to opioid receptor antagonists	No increase in LH and PRL	Small increase in LH and no increase in PRL [78,79]	Increase in LH [80]^h^

^a^Common traits of pseudocyesis, PCOS and/or major depressive disorder are highlighted using bold text.

^b^Although the true incidence of galactorrhea in PCOS remains unclear and is probably only 1 or 2%, a study published in 1980 [81] reported that ≈ 24% of PCOS women show galactorrhea irrespectively of the concomitant presence of either hyperprolactinemia (≈14%) or normoprolactinemia (≈10%).

^c^PCOS women have increased GnRH pulse frequency because of androgen-mediated decreased GnRH sensitivity to P feedback inhibition. The higher GnRH pulse frequency increases LH pulsatility and favors LH production over FSH (for reviews, see McCartney et al. [36] and Burt-Solorzano et al. [59]).

^d^Whereas untreated premenopausal women suffering from major depression display E_2_ levels on the first day after menstruation similar to those found in healthy age-matched control women [62], they often have plasma levels of E_2_ in the follicular phase significantly lower than control healthy women (for reviews, see Swaab et al. [82] and Williams et al. [60]), although they exhibit higher amplitudes of diurnal E_2_ rhythms [83].

^e^Levels in PCOS women show the typical changes associated with either anovulation (P deficiency, i.e., within the follicular phase range) or ovulation (within the luteal phase range) [84,85]. Note that nearly 12% of oligo- or amenorrheic PCOS women show signs of spontaneous ovulation based on random P assessment [86].

^f^Some of the discrepancies between cortisol and ACTH responses to dexamethasone in major depressed individuals may be due to the low sensitivity and specificity of the ACTH assays used in some studies [42].

^g^The hyperandrogenemia in PCOS may contribute to the relatively reduced response of GH to dopamine agonists (for review, see Spiliotis [49]).

^h^The study by Martín del Campo et al. [80] is methodologically flawed. In particular, they analyzed a heterogeneous group of women with major depression (4 post-menopausic, 2 eumenorrheic and one with irregular cycles) and did not control for the phase of the menstrual cycle. In addition, they used both women and men as control subjects. Despite these methodological deficiencies, there is evidence of dysregulated endogenous opioid emotion regulation circuitry in women with major depressive disorder [87].

### Neuroendocrine/endocrine pathways of pseudocyesis

Most pseudocyetic women suffer from mild to major depression, anxiety and/or emotional stress due to psychologic conflicts or needs, such as women who simultaneously wish for children and fear becoming pregnant (for reviews, see Small [31], O’Grady and Rosenthal [9] and Whelan and Stewart [88]) or women who have an overwhelming desire to become pregnant because of societal pressures [3,7]. In this context, we should mention that patients with major depression have a deficit in brain dopamine and norepinephrine activity [89], and increased sympathetic nervous system activity associated with a higher rate of entry to plasma of norepinephrine released from sympathetic nerves (norepinephrine spillover rate) [90,91] and elevated plasma levels of norepinephrine [92]. In rodents, chronic psychosocial stress is also associated with reduced brain dopamine and norepinephrine activity and elevated plasma levels of norepinephrine (for reviews, see Goddard et al. [93] and Rasheed and Alghasham [94]). PCOS women also show low dopamine hypothalamic tone (for review, see Hernández et al. [95]).

Data from the present review indicate that pseudocyetic women may have a deficit in brain dopamine activity. This fact supports the notion that pseudocyetic women may have a dysfunction of central nervous system catecholaminergic pathways involved in the regulation of anterior pituitary hormone secretion. Dopamine [96] and norepinephrine [97] have been recently identified in mice as potent steroid-independent inhibitors of gonadotropin-releasing hormone (GnRH) neuron excitability and firing. Moreover, dopamine in women inhibits pulsatile LH (for review, see Jaffe et al. [98]) and PRL (for review, see Ben-Jonathan and Hnasko [48]) secretion. Thus, a deficit/dysfunction in brain catecholaminergic activity may result in increased pulsatile GnRH, LH and PRL and an elevated LH/FSH ratio. These endocrine changes may induce hypomenorrhea or amenorrhea, galactorrhea and diurnal and/or nocturnal hyperprolactinemia - traits found in most pseudocyetic women. Also, the reduced brain catecholaminergic activity displayed by pseudocyetic women may combine/interact with a drop in steroid feedback inhibition of GnRH. In particular, the relatively high levels of T found in pseudocyetic women may decrease GnRH sensitivity to P feedback inhibition resulting in increased GnRH pulse frequency, elevated LH pulsatility and higher LH production over FSH such as occurs in PCOS women (for reviews, see McCartney et al. [36] and Burt-Solorzano et al. [59]). Finally, the increased sympathetic nervous system activity likely shown by pseudocyetic women may be implicated, not only in the apparent fetal movements and labor pains at the expected date of delivery felt by some pseudocyetic women, but also in the typical abdominal enlargement displayed by most pseudocyetic women.

Several authors have proposed a mixture of causes to explain the physiological mechanism by which abdominal swelling takes place in pseudocyetic women. This mixture of causes includes chronic contraction of the diaphragmatic muscle (this contraction pushes the bowel downward in the abdominal cavity), assumption of a lordotic posture, increased omental and abdominal wall fat, and mild to marked constipation and/or bowel distention (for reviews, see Small [31], O’Grady and Rosenthal [9] and Whelan and Stewart [88]). However, the distended abdomen, that may remain bloated for months immediately (within minutes or even seconds) disappears, with or without release of flatus, after pseudocyetic women are convinced of their non-pregnant state or under anesthesia. This observation indicates that although increased omental and abdominal wall fat, and mild to marked constipation and/or bowel distention may contribute to abdominal enlargement, they are not primary causes of abdominal protrusion. Furthermore, unlike normal pregnancy, the umbilicus does not become everted, the abdominal distension is uniform and rounded, and the abdominal wall presents a rubbery, tense muscular tone being tympanic on percussion. All these facts point to the diaphragm and/or certain groups of muscles of the abdominal wall as the primary cause of abdominal enlargement. In particular, pseudocyetic women may experience chronic contraction of the diaphragm accompanied by abnormal patterns of contraction/relaxation of the anterior abdominal and internal oblique muscles. This mechanism, called *abdomino-phrenic dyssynergia*, has been observed in patients with abdominal bloating and distension after colonic-gas-load-induced abdominal distension [99]. Interestingly, literature shows cases of persistent [100] or spasmodic (spasms resembling those of a woman in labor [101]) *hysterical abdominal proptosis* likely resulting from abdomino-phrenic dyssynergia.

It is worth mentioning that, in contrast to normal individuals that can push out the abdominal wall only for a relatively short period of time even in the presence of the quietest respiration, persistent hysterical abdominal proptosis is unaffected by a cough or by a sneeze, nor by emptying the bladder or by straining at stool [100]. However, it disappears after relaxing the patient’s diaphragm by having the patient take a deep inspiration followed by a sudden expiration or by having the patient hold her/his breath as long as possible such as occurs in pseudocyesis (for reviews, see Alvarez [102] and O’Grady and Rosenthal [9]).

Importantly, the 2 phrenic nerves that originate in the neck (mainly from the 4^th^ cervical nerve, but which also receive contributions from the 5^th^ and 3^rd^ cervical nerves) and pass down between the lung and heart to reach the diaphragm, contain not only motor fibers but also proprioceptive and sympathetic fibers to the diaphragm, and sensory fibers to the pleura and pericardium. Therefore, dysfunction of the sympathetic nervous system in pseudocyetic women, mediated directly via impulses transmitted through the sympathetic nervous system and/or indirectly via plasma norepinephrine released from sympathetic nerves or secreted from the adrenal medulla, may induce abdomino-phrenic dyssynergia resulting in abdominal protrusion.

## Concluding remarks

In this literature review, we have analyzed epidemiological, psychiatric/psychologic, gynecological and endocrine traits of 10 pseudocyetic women reported in 5 selected studies after applying stringent criteria to discriminate between cases of pseudocyesis vera versus delusional, simulated or erroneous pseudocyesis. The analysis performed shows that pseudocyetic women share many endocrine traits with PCOS and major depressive disorder, although these traits are more akin to PCOS than to major depressive disorder.

The reviewed data support the notion that pseudocyetic women may have: increased sympathetic nervous system activity; dysfunction of central nervous system catecholaminergic pathways involved in the regulation of hormone secretion from adenohypophysis; and decreased steroid feedback inhibition of GnRH. These neuroendocrine/endocrine disorders may cause hypomenorrhea or amenorrhea, galactorrhea, diurnal and/or nocturnal hyperprolactinemia, abdominal distension and apparent fetal movements and labor pains at the expected date of delivery - traits exhibited by most pseudocyetic women. However, other neuroendocrine/endocrine pathways not yet analyzed in pseudocyetic women may also be involved in the development of pseudocyetic traits. These may include, the hypothalamic-pituitary-adrenal axis; neurotransmitters controlling GnRH neuron excitability [96]; and dopamine stimulators and inhibitors of PRL secretion (for review, see Ben-Jonathan and Hnasko [48]).

## Abbreviations

ACTH: Adrenocorticotropin hormone; CCK: Cholecystokinin; DHEAS: Dehydroepiandrosterone sulfate; E2: Estradiol; EB: Estradiol benzoate; FAI: Free androgen index; FSH: Follicle stimulating hormone; GABA: γ-aminobutyric acid; GH: Growth hormone; GHIH: GH-inhibiting hormone; GHRH: GH-releasing hormone; GnRH: Gonadotropin-releasing hormone; L-DOPA: L-3,4-dihydroxyphenylalanine; LH: Luteinizing hormone; P: Progesterone; PRL: Prolactin; PCOS: Polycystic ovarian syndrome; SEM: Standard error of the mean; SRIF: Somatotropin release-inhibiting factor; T: Testosterone; T4: Thyroxine; TSH: Thyroid-stimulating hormone; TRH: Thyrotropin-releasing hormone.

## Competing interests

The authors declare that they have no competing interests.

## Authors’ contributions

JJT was involved in the conception and design of the study, the acquisition, analysis and interpretation of data and drafting of the article. CH, MAGP and AC were involved in the analysis and interpretation of data, and revising the article critically for important intellectual content. All authors read and approved the final manuscript.
